# Successful anesthetic management during abdominal wall-lifting laparoscopic cholecystectomy in a patient with hereditary angioedema

**DOI:** 10.1186/s40981-018-0174-1

**Published:** 2018-05-09

**Authors:** Masashi Fujii, Takayuki Higashiguchi, Nobuaki Shime, Yasuyo Kawabata

**Affiliations:** 1 Department of Anaesthesia, Nagahama Red Cross Hospital, 14-7 Miyamae-cho, Nagahama, Shiga 526-8585 Japan; 2 Department of Surgery, Nagahama Red Cross Hospital, Nagahama, Shiga Japan; 3 Department of Emergency and Critical Care Medicine, Institute of Biomedical & Health Sciences, Hiroshima University, Hiroshima, Japan

**Keywords:** Hereditary angioedema, Wall-lifting laparoscopy, Cholecystectomy, Spinal-epidural anesthesia

## Abstract

**Background:**

Hereditary angioedema is a rare genetic disorder resulting from an inherited deficiency or dysfunction of the C1-esterase inhibitor. In the anesthetic management of such patients, special caution should be exercised while attempting tracheal intubation because it may cause mucosal edema in the upper airway.

**Case presentation:**

A 52-year-old female with hereditary angioedema was scheduled for laparoscopic cholecystectomy. C1-esterase inhibitor, Danazol, tranexamic acid, and prednisolone were administered on the day of surgery. An epidural catheter was inserted through the intervertebral space at T9/10, and spinal anesthesia was instilled via the L3/4 intervertebral space. A single-hole, Nishii-type lifting laparoscopic surgery, without pneumoperitoneum (i.e., gasless) was completed uneventfully.

**Conclusion:**

This report described the successful management of a patient with hereditary angioedema who underwent laparoscopic cholecystectomy using spinal-epidural anesthesia without tracheal intubation and lift type laparoscopic surgery. This approach to anesthetic management could be indicated in cases with a similar presentation.

## Background

Hereditary angioedema (HAE) is a rare genetic disorder resulting from an inherited deficiency or dysfunction of the C1-esterase inhibitor and has a reported incidence of between one in 10,000 and one in 50,000 of the total population worldwide [1, 2]. The main symptoms of HAE include subcutaneous or mucosal edema, abdominal pain, nausea, vomiting, or upper airway obstruction [3]. After dental or intraoral surgery, or endotracheal intubation, the risk for upper airway edema due to direct mucosal stress is a concern [4, 5]. It is advisable to avoid tracheal intubation, if possible, because the mortality rate due to upper airway obstruction in HAE has been reported to be as high as 30–50% if not properly addressed [6]. Combined spinal-epidural anesthesia is an important option for reducing intubation-associated complications. Laparoscopic gallbladder surgery with pneumoperitoneum using carbon dioxide could be performed using spinal or epidural anesthesia [7–9]. However, radiation pain to the right shoulder caused by the stimulation of the diaphragm by pneumoperitoneum with carbon dioxide or anxiety of the patient becomes a risk factor for transitioning to general anesthesia [7–10]. In the present case, single-hole Nishii-type lifting laparoscopic surgery was performed without pneumoperitoneum [11, 12] and did not involve carbon dioxide; therefore, the greatest cause for conversion to general anesthesia was avoided. We report a case with HAE which was successfully managed using spinal-epidural anesthesia during abdominal wall-lifting laparoscopic gallbladder surgery.

## Case presentation

The patient provided informed consent for the procedure and the publication of anonymized case details presented in this report.

A 52-year-old woman (height, 155 cm; weight, 64 kg) with HAE was scheduled for laparoscopic cholecystectomy. An immunology clinic confirmed the presence of HAE at the age of 39, and the patient was diagnosed with type-I HAE according to genetic testing. Tranexamic acid 1000 mg and danazol 100 mg had been administered daily for long-term symptom control. In addition, prednisolone 4 mg was orally administered due suspected systemic lupus erythematosus. Preoperative blood tests revealed mild liver dysfunction (total bilirubin, 1.9 mg/dL; aspartate aminotransferase, 41 IU/L; alanine aminotransferase, 75 IU/L). Chest X-ray imaging, electrocardiography, and respiratory function tests were normal.

Previously, the patient had repeatedly developed signs of angioedema at a frequency of once every 2–3 months. Nasal obstruction, edema around the cervical region, or a feeling of discomfort in the throat due to angioedema were reported, necessitating emergency infusion with C1-esterase inhibitor concentrate (Berinert® P, CSL Behring, Germany) to relieve the symptoms.

During preoperative planning of anesthesia for the current surgery, avoiding tracheal intubation to reduce the risk for tracheal angioedema was considered. Wall-lifting laparoscopic surgery was scheduled so that surgical stress could be managed using combined spinal-epidural anesthesia.

Danazol, tranexamic acid, and prednisolone were administered orally on the morning of the day of surgery; in addition, 1500 U of C1-esterase inhibitor was administered intravenously 2 h before the surgery.

An epidural catheter was inserted through the intervertebral space at T9/10, and spinal anesthesia was instilled using 0.5% hyperbaric bupivacaine 3 mL and fentanyl 15 μg via the L3/4 intervertebral space. In addition, 5 mL of 0.375% levobupivacaine solution and 3 mg of morphine were administered via the epidural catheter. Anesthesia was achieved below the T4 cutaneous level. In accordance with the patient’s request, she was sedated by intermittent administration of midazolam (7 mg total) and continuous infusion of propofol (0.8–1.6 mg/kg/h). Oxygen was administered at 4 l/min via a face mask, and peripheral oxygen saturation was maintained at 100% through the operation. Blood pressure was maintained by intermittent administration of phenylephrine. A single-hole, Nishii-type lifting laparoscopic surgery, without pneumoperitoneum (i.e., gasless) was completed uneventfully. The operation duration was 2 h and 45 min, and anesthesia duration was 3 h and 20 min. After confirming the absence of complications in respiratory status, she was returned to the ward and discharged uneventfully on postoperative day 3.

## Discussion

To the best of our knowledge, this is the first report of abdominal wall-lifting laparoscopic cholecystectomy in a patient with hereditary angioedema. Laparoscopic gallbladder surgery, with pneumoperitoneum using carbon dioxide can be performed using spinal or epidural anesthesia [7–9]. However, radiating pain to the right shoulder, caused by the stimulation of the diaphragm by pneumoperitoneum with carbon dioxide or patient anxiety, is a risk factor for transitioning to general anesthesia [7–10]. Referred pain caused by pneumoperitoneum may be induced by irritation of the phrenic nerve originating from the third to the fifth cervical nerves [13] and peritoneum innervated by the third to the sixth thoracic nerves [14–16], suggesting that analgesia level up to the cervical region is required to eliminate it. In the present case, single-hole Nishii-type lifting laparoscopic surgery was performed without pneumoperitoneum [11, 12] and did not involve carbon dioxide; therefore, the greatest cause for conversion to general anesthesia was avoided. Moreover, non-gas laparoscopic cholecystectomy may require a lower level of anesthesia than cholecystectomy with pneumoperitoneum. The lower level of spinal-epidural anesthesia may contribute to reducing the risk for respiratory depression and avoidance of conversion to general anesthesia.

Airway edema in HAE cannot be predicted by the frequency of or intervals between attacks [17] and, as such, pharmacological prophylaxis is desirable [18]. In accordance with the established World Allergy Organization guideline [19], we administered 1500 U of C1-esterase inhibitor, which was prepared in the operating room for additional use, in addition to danazol and tranexamic acid, preoperatively.

Herein, we described the successful management of a patient with HAE who underwent laparoscopic cholecystectomy using spinal-epidural anesthesia without tracheal intubation and lift-type laparoscopic surgery. This approach to anesthetic management could be indicated in cases with a similar presentation.

## Abbreviations

C: Cervical
HAE: Hereditary angioedema
L: Lumbar
T: Thoracic
